# Density of routinely collected neurology data depends on patient visit type: an investigation using the observational medical outcomes partnership common data model

**DOI:** 10.1136/bmjno-2025-001202

**Published:** 2025-09-23

**Authors:** Fran Biggin, Laura M White, Quinta Ashcroft, Timothy Howcroft, Vishnu Vardhan Chandrabalan, Hedley Emsley, Jo Knight

**Affiliations:** 1 Lancaster University Faculty of Health and Medicine, Lancaster, UK; 2 Lancashire Teaching Hospitals NHS Foundation Trust, Preston, UK

**Keywords:** HEALTH POLICY & PRACTICE, STATISTICS, CLINICAL NEUROLOGY

## Abstract

**Background:**

The Observational Medical Outcomes Partnership (OMOP) Common Data Model (CDM) is a standardised framework for organising healthcare data. This study uses data in the OMOP CDM format to analyse information on neurology patients.

**Methods:**

Routinely collected data harmonised to OMOP at a large referral hospital in England were used. A study cohort was defined as patients who attended at least one neurology outpatient appointment between 01 April 2022 and 31 March 2023 (n=23 862). Data collected at all visits to the hospital made by this cohort between 01 April 2021 and 31 March 2024 were extracted. The cohort was then divided into four subcohorts according to appointment types attended: outpatient appointment(s) only (n=15 2); outpatient appointment(s) and inpatient stay(s) (n=2750); outpatient appointment(s) and emergency department attendance(s) (n=1658); outpatient appointment(s), inpatient stay(s) and emergency department attendance(s) (n=4199).

**Results:**

We found there to be more data available for patients who had at least one inpatient stay or emergency department attendance than for those with only outpatient appointments. Notably, an average of 0 out of 100 patients in the outpatient only subcohort had a record of a condition, compared with 100 out of 100 patients in the subcohort with outpatient appointments, emergency attendances and inpatient stays.

**Conclusions:**

Neurology outpatients have far less data recorded than inpatients or patients attending emergency departments. This disparity arises from the lack of outpatient diagnostic coding and impairs the advancement of research in this area. Using the OMOP CDM structure makes it easy to highlight these differences.

**WHAT IS ALREADY KNOWN ON THIS TOPIC**

- The Observational Medical Outcomes Partnership (OMOP) common data model (CDM) is being adopted by the National Health Service (NHS) to provide a uniform structure to the data within the NHS Secure Data Environments to support research. We know that outpatient coding is not mandated, so diagnoses at outpatient appointments are not regularly recorded in electronic health records.

**WHAT THIS STUDY ADDS**

- We investigate the variable volume of data available for research through a secondary care dataset that has been converted to the OMOP CDM. We show that outpatients have far less data recorded than inpatients or patients attending ED, in terms of both volume and type of data.

**HOW THIS STUDY MIGHT AFFECT RESEARCH, PRACTICE OR POLICY**

- This study highlights the need for data systems such as Secure Data Environments to be based on data which are complete. We also highlight the importance of ensuring that data recording for outpatients is as complete as it is for inpatients and ED.

## Introduction

 Healthcare data are large and are collected for many different reasons ranging from administration and financing to diagnosis and treatment. In the UK, healthcare is delivered by the National Health Service (NHS). The structure of the NHS is complex, with different Trusts (organisations that manage NHS hospitals and healthcare services in a specific area) commissioning services independently. This has created a situation in which there are a multitude of different electronic systems and databases in use both across and within Trusts. This creates a fractured data landscape where data in one hospital are not compatible with data from another, leading to problems when trying to manage patients who have used services from different Trusts, and when trying to amalgamate data for research purposes. One potential approach to solving this problem is the use of Common Data Models (CDMs) which create standardised structures for representing and storing data, allowing for common understanding of concepts and the ability to share data and processes across institutions that use the same model.

The Observational Medical Outcomes Partnership CDM (OMOP CDM) is a data model designed to be used for observational health data.^1^ It provides a framework for transforming data from electronic health records and other sources into a set of standard vocabularies stored in a standardised database structure.^2^ This standardisation allows for the creation of multipartner studies across geographical boundaries as institutions can share analysis pipelines instead of data.^3^ It is currently used by a number of institutions across the USA, Europe and Asia^4^ and the Observational Health Data Sciences and Informatics (OHDSI) collaborative, who maintain the OMOP CDM standards, have 4294 collaborators across 83 countries.^5^ The OMOP CDM has been chosen by the NHS to form the structure of their network of Secure Data Environments (SDEs).^6^ The SDEs are trusted research environments designed to offer approved users secure, controlled access to pseudonymised healthcare data under strict rules of information governance.

The North West SDE covers the NHS Integrated Care Systems (ICS) of Cheshire and Merseyside, Greater Manchester and Lancashire and South Cumbria (L&SC). Lancashire Teaching Hospitals NHS Foundation Trust (LTHFT), which is part of L&SC ICS, has been instrumental in creating infrastructure and transforming data for the North West SDE^7^ as well as for the Lancashire and South Cumbria SDE.^8^ Several of the Trust’s databases containing patient data have been transformed into the OMOP CDM format. In this study, we focus on the Trust’s data for neurology outpatients (OPs) that have been transformed into the OMOP CDM.

Neurology data have been a focus of interest for LTHTR for the past 5 years and previous studies have shown that neurology OPs lack diagnostic coding.^9^ Since many neurological diseases are chronic in nature,^10^ a high proportion of patients require long-term monitoring in the OP setting. The paucity of diagnostic coding for neurology patients who are seen solely in OP clinics creates a barrier for clinical research and impairs effective hospital resource planning for OP services. A lack of OP diagnostic coding has also been identified for neurology patients in other UK Trusts^11^ and for OPs in other medical specialties,^12 13^ in particular, paediatrics.^14^

In this study, we aim to quantify the volume and type of data available for neurology OP appointments, especially when compared with other types of visit (inpatient (IP) stays and emergency department (ED) attendances). It is important to understand what types of data are currently available as this can tell us what research is currently possible and what data may need to be brought into the OMOP CDM database to facilitate future studies and potential clinical uses.

## Methodology

This study draws on data from the LTHFT. This trust comprises two acute hospitals providing around 1000 beds covering a local population of around 400 000. The trust also provides a regional neurosciences service covering a geographically and socioeconomically diverse population of approximately 1.6 million. Patients can be referred into the neurology services from primary care (eg, from a general practitioner), as an emergency referral from the ED or as an internal referral from other hospital specialties.

Between March and October 2023, several LTHFT databases were transformed into the OMOP CDM format through a detailed extract-transform-load (ETL) process.^15^ All our ETL documentation can be found on GitHub (https://lsc-sde.github.io/idril-docs). Not all data from the electronic health record (EHR) were harmonised to the OMOP CDM as a balance needed to be struck between the volume of data to be harmonised and the complexity of mapping all terms including very infrequently used data fields. However, the rarely used fields that were not harmonised are unlikely to have had an impact on this study. The resulting database was assessed using the OHDSI Data Quality Dashboard tools. The database was also validated by Health Data Research UK (HDRUK) for inclusion in their data gateway and by an independent, European Health Data Evidence Network (EHDEN) certified partner as a prerequisite to entry into the EHDEN catalogue. Data harmonisation was completed using internal data engineering resources with additional funding from an EHDEN-HDRUK data partner funding call. There are no licensing costs for the OMOP CDM.

This research makes use of a pseudonymised instance of the LTHFT OMOP database IDRIL-1 (Incrementing Database for Research in Lancashire),^16^ and the data were extracted in April 2024.

All relational databases are structured around the use of tables, with common fields in those tables allowing them to be linked to each other. The OMOP CDM is made up of several standardised tables based on categories of data^17^ and uses several fields such as ‘person_id’ or ‘concept_id’ which enable the tables to be linked. The ‘visit occurrence’ table contains administrative information on visits such as when visits occurred, the type of visit and the location of the visit. The tables which contain the majority of the clinical information are ‘condition occurrence’, ‘drug exposure’, ‘procedure occurrence’, ‘measurement’ and ‘observation’. A description of the OMOP tables used in this study can be found in table 1; descriptions are taken from the OHDSI website.^17^

**Table 1:** A description of the OMOP tables used in this study

OMOP table name/data category name as used in this study	Description	Example information contained in table
condition_occurrence/condition	This table contains records suggesting the presence of a disease or medical condition stated as a diagnosis, a sign or a symptom, which is either observed by a provider or reported by the patient.	A diagnosis of epilepsy.
drug_exposure/drug	This table captures records about the exposure to a drug ingested or otherwise introduced into the body	A prescription for levetiracetam.
procedure_occurrence/procedure	This table contains records of activities or processes ordered by, or carried out by, a healthcare provider on the patient with a diagnostic or therapeutic purpose.	A record of an MRI brain.
Measurement/measurement	This table contains records of measurements, that is, structured values (numerical or categorical) obtained through systematic and standardised examination or testing of a person or person’s sample.	The results of a complete blood count.
Observation/observation	This table captures clinical facts about a person obtained in the context of examination, questioning or a procedure.	A record that the patient has a family history of stroke.
visit_occurrence/visit	This table contains events where persons engage with the healthcare system for a duration of time.	A record of an outpatient appointment with a consultant neurologist.

OMOP, Observational Medical Outcomes Partnership.

We identified a cohort of neurology OPs using the ‘visit occurrence’ table. We searched for all patients with an OP clinic appointment between 01 April 22 and 31 March 23 where the ‘visit source value’ was ‘OP’, and the ‘provider id’ contained an id from a list of known neurology specialists. We then extracted all data recorded for these patients between 01 April 21 and 31 March 24 (1 year preceding the start of and 1 year following the end of the cohort identification period) from the ‘condition occurrence’, ‘drug exposure’, ‘procedure occurrence’, ‘measurement’ and ‘observation’ tables. Note that all patients have had at least one neurology OP appointment during the study period, but that data on visits for all reasons were extracted for analysis. The overall cohort is one of neurology OPs, but we include in the analysis hospital visits for any and all reasons, not just neurology.

We created four subcohorts from the initial cohort of neurology OPs:

1. OP appointments only (OP only): patients that have only had OP appointments and no other visit types during the study period 01 April 21 to 31 March 24.
2. OP and IP: patients that have had at least one OP appointment and at least one IP stay but no ED attendances during the study period 01 April 21 to 31 March 24.
3. OP and ED: patients who have had at least one OP appointment and at least one ED attendance but no IP stay during the study period 01 April 21 to 31 March 24.
4. OP, IP and ED: patients who have had at least one neurology OP appointment and at least one IP stay and at least one ED attendance visit during the study period 01 April 21 to 31 March 24.

We used these subcohorts to examine the differences in the volume and type of information available on different groups of patients. We examined the overall volume of data available of different data categories, the median number of records created during a visit-day and the percentage of patients who could be expected to have at least one record in a particular category of data.

In order to compare the number of records per visit, we use the concept of a ‘visit-day' to ensure that we are accounting for visits that occur over multiple days. A ‘visit-day’ is 1 day of a visit, so an OP appointment that occurs on 1 day accounts for one visit-day, and an IP stay with one overnight occurs over two visit-days. Note that many IP stays are day cases and are therefore only accounted for one visit-day (eg, for dialysis or chemotherapy).

### Public patient involvement statement

No public and patient involvement.

## Results

We found 23 862 patients who had at least one neurology OP appointment between 01 April 22 and 31 March 23. Between 01 April 21 and 31 March 24 these patients had a total of 262 592 hospital visits, split between OP appointments (205 180), IP stays (36 631) and ED attendances (20 781). These patients are split into four subcohorts for further analysis depending on the types of visit they made during the study period (see table 2). We can see that the largest subcohort is the group of patients who only visited the hospital for an OP appointment, with the 15 244 patients in this subcohort accounting for 64% of the total study population.

**Table 2:** Size of the subcohorts

Sub-cohort name	Number of patients	Percentage of study population
Outpatient appointments only	15 255	63.88%
Outpatient appointments and inpatient stays	2750	11.52%
Outpatient appointments and ED attendances	1658	6.95%
Outpatient appointments, inpatient stays and ED attendances	4199	17.60%

ED, emergency department.

Table 3 shows the number of individual records present in each of the five data categories for each of the four subcohorts. We can see from this table that subcohort 4 (OP, IP and ED) accounts for 80.7% of all the records we retrieved despite it representing only 18% of the study population (over 11 million records for 4199 patients). Within this subcohort, the drugs and measurement categories are the two with the highest percentage representation (85.7% and 82.7% of all records). In contrast, subcohort 1 (OP only) represents only 1.3% of the total number of records, despite being the largest subcohort.

**Table 3:** Number of records per subcohort in each of the OMOP tables

Subcohort	Condition (%)	Drug (%)	Measurement (%)	Observation (%)	Procedure (%)	Total (%)	Mean records per person
1 (OP only) n=15 244	53 (0.02)	2799 (0.2)	143 391 (1.4)	34 650 (1.3)	7068 (5.1)	187 961 (1.3)	12.3
2 (OP and ED) n=1658	2943 (0.98)	605 (0.1)	136 685 (1.3)	19 504 (0.7)	6553 (4.8)	166 290 (1.1)	100.3
3 (OP and IP) n=2750	72 306 (24.5)	166 077 (14.0)	1 506 428 (14.6)	682 122 (25.9)	29 718 (21.7)	2 456 651 (16.9)	893.3
4 (OP, IP and ED) n=4199	219 708 (74.5)	1 017 045 (85.7)	8 506 073 (82.7)	1 899 813 (72.1)	93 798 (68.4)	11 736 437 (80.7)	2795.1
**Total n=23 862**	**295 010**	**1 186 526**	**10 292 577**	**2 636 089**	**137 137**	**14 547 339**	**609.6**

Note: percentages relate to the total numbers of records in each category, that is, the column.

ED, emergency department; IP, inpatient; OP, outpatient.

Table 4 shows the number of hospital visits made by patients in each of the four subcohorts. We can see that patients in subcohort 4 (OP, IP and ED) had the highest number of all types of visit, including more OP appointments than subcohort 1 (OP only) despite the subcohort being a third of the size. This may be due to the fact that patients who require both IP stays and ED attendances may have more complex conditions and multiple comorbidities, leading to the need for a higher number of OP appointments. All patients who had ED attendances that led to IP stays are included in subcohort 4. There are 5520 of these types of visit and, for the purpose of this analysis, they are counted twice, both as an ED attendance and as an IP stay.

**Table 4:** Number of visits attended

Subcohort	OP appointments	IP stays	Emergency attendances	Total
1 (OP only) n=15 255	77 054	0	0	77 054
2 (OP and IP) n=2750	34 653	14 650	0	49 303
3 (OP and ED) n=1658	14 379	0	3269	17 648
4 (OP, IP and ED) n=4199	79 094	21 981*	17 512*	118 587
**Total n=23 862**	**205 180**	**36 631**	**20 781**	**262 592**

* The 5520 visits that encompass an ED attendance and an inpatient stay are included in both these cells.

ED, emergency department; IP, inpatient; OP, outpatient.

**Figure 1:**
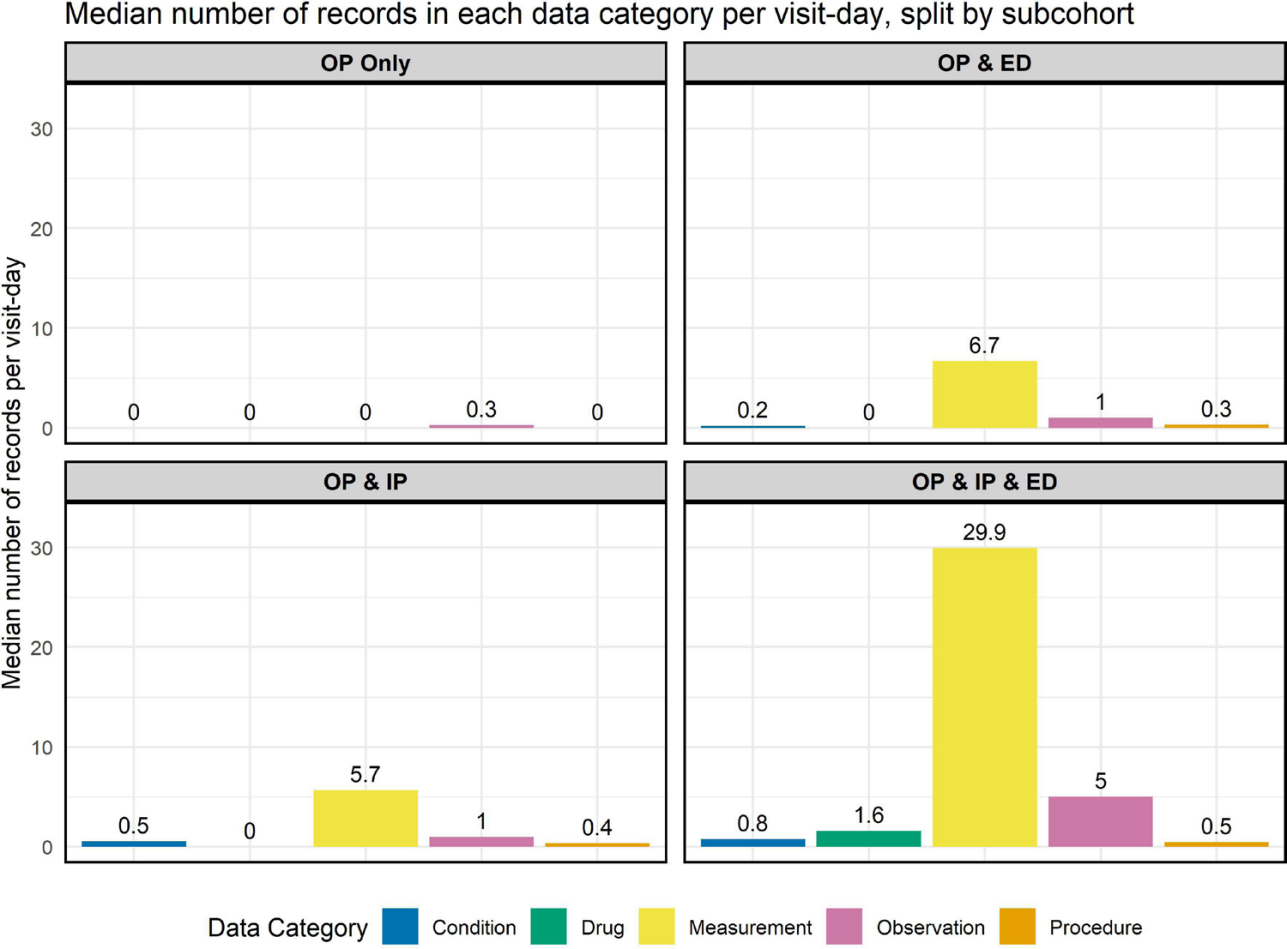
Bar chart showing the median number of records made per visit-day in each of the data categories, per subcohort. ED, emergency department; IP, inpatient; OP, outpatient.

In table 5, we show the median number of records made per visit day. We see that sub-cohort 4 (OP, IP and ED) again receives the largest number of records in all five categories. Patients in this subcohort receive a median of 29.90 measurement records per visit-day. In contrast, patients in the OP only subcohort on average only receive records in the observations table at a median rate of 0.27 per visit-day. This difference may occur because IPs are monitored throughout a stay with the recurrent capture of physiological measures such as blood pressure. The disparity becomes clear when we examine figure 1. It is also worth noting that, despite also including IP stays, subcohort 3 (OP and IP) only has a median of 5.68 records per visit-day in contrast to 29.90 in subcohort 4 (OP, IP and ED). This may be accounted for by the fact that the median length of an IP stay for patients in subcohort 3 is 1 day, whereas in subcohort 4 it is 3 days. If a patient has an IP stay of only 1 day, for example, as a day case for dialysis or chemotherapy, they are less likely to generate a large number of measurements during their stay.

**Table 5:** Median number of records made per visit-day

Subcohort	Condition	Drug	Measurement	Observation	Procedure
1 (OP only) n=15 255	0.00	0.00	0.00	0.27	0.00
2 (OP and ED) n=1658	0.20	0.00	6.68	1.00	0.33
3 (OP and IP) n=2750	0.53	0.00	5.68	1.00	0.36
4 (O, IP and ED) n=4199	0.75	1.60	29.90	5.03	0.47

ED, emergency department; IP, inpatient; OP, outpatient.

**Figure 2:**
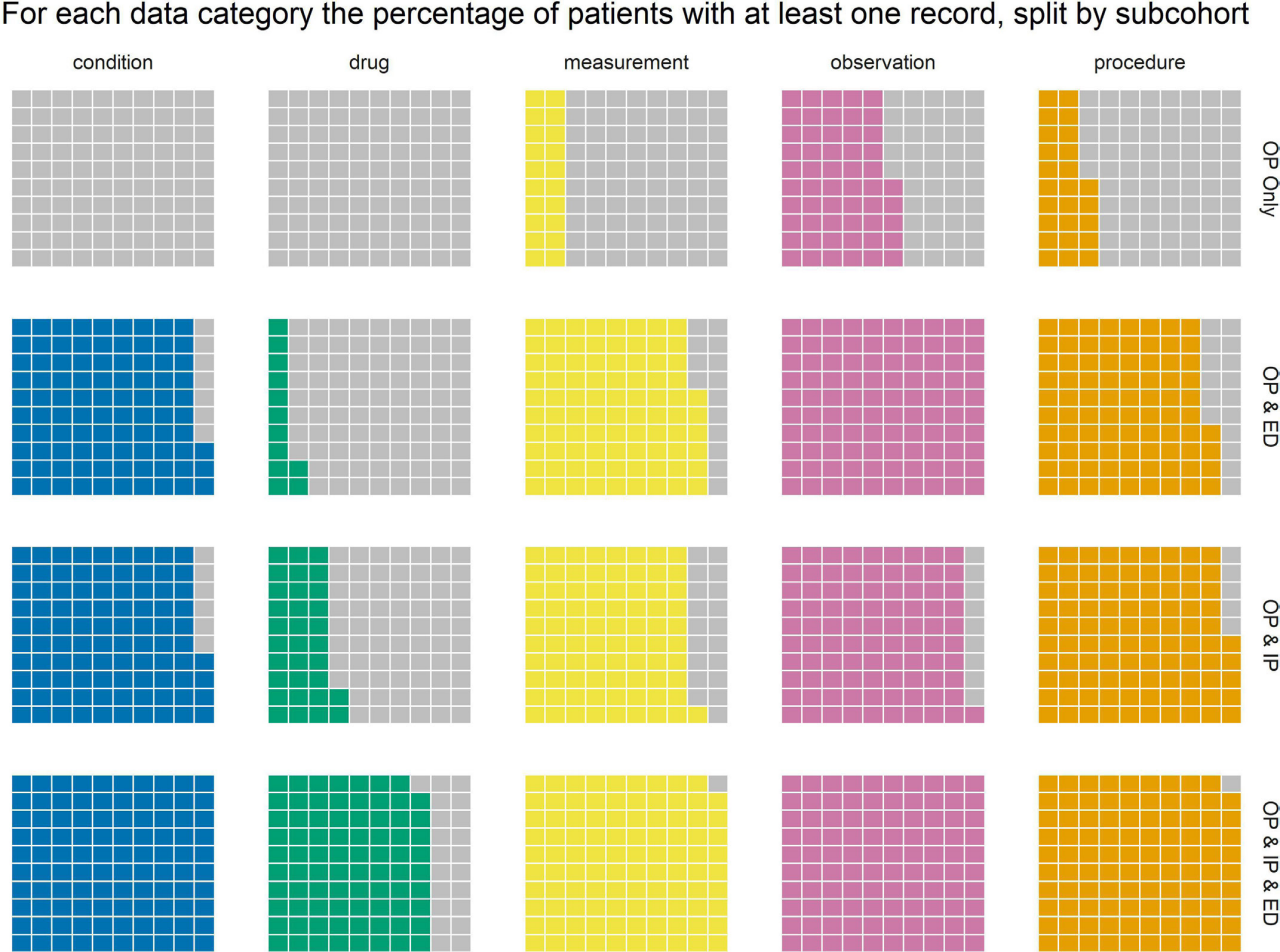
A waffle chart showing for each data category, the percentage of patients who have at least one record of that type. ED, emergency department; IP, inpatient; OP, outpatient.

Table 6 shows, for each data category, the percentage of patients who have at least one record of that type. This is visualised as a waffle chart in figure 2. This clearly shows that the OP only sub-cohort is almost entirely lacking any records for conditions and drugs, meaning that a clinician will not know, from the currently OMOP-mapped medical records, if a patient in this subcohort has a prior diagnosis or prescription. In contrast, 99.69% patients in subcohort 4 (OP, IP and ED) have at least one condition recorded, and 78.66% have at least one record of a prescription. The percentages for conditions, measurements, observations and procedures are also high for subcohorts 2 and 3.

**Table 6:** For each data category, the percentage of patients who have at least one record of that type

Subcohort	Condition	Drug	Measurement	Observation	Procedure
1 (OP Only)	0.02	0.06	20.24	55.02	24.68
2 (OP and ED)	92.82	11.94	85.83	100.00	83.72
3 (OP and IP)	94.18	31.64	80.55	91.38	94.76
4 (OP, IP and ED)	99.69	78.66	99.33	100.00	99.43

ED, emergency department; IP, inpatient; OP, outpatient.

## Discussion

We have undertaken an investigation of data available on neurology patients using the OMOP CDM. We have quantified the volume and type of data available for neurology OP appointments and compared this to other visit types, and this has helped us to understand what types of data are currently available. Using data in the OMOP CDM format allowed us to make a clear and structured analysis of a range of different types of data. The results show a stark contrast between the large volume of data collected for patients with IP stays or ED visits and the paucity of data for patients who only attend the hospital for OP appointments. This is in part due to the fact that patients who attend only OPs have fewer individual attendances than those who also visit as IPs and/or attend ED. However, patients who attend only OP appointments also have far fewer records made per visit-day and have almost zero records regarding conditions or drugs. This leads to a large imbalance in the information available on a patient which depends on what type of appointments they have previously attended.

The virtual absence of information on conditions or drugs for OPs is of great concern. Knowledge of previous diagnoses and comorbidities, past and current prescribed medications, is a key piece of information required by a clinician at an OP appointment. Currently, this information needs to be gleaned from the patient at the appointment, as it is not available in the data. This reduplicates information requests from patients and further pressurises scarce clinician time.

Furthermore, bias is introduced by virtue of the fact that when extracting patients with a particular diagnosis from the healthcare records for research purposes, only those who have required an ED visit or IP admission will have the diagnostic code in their record. The patient population will therefore include those who have more severe neurological disease or complex comorbidities that require frequent ED and IP visits, while patients with milder neurological disease that is managed effectively in OP clinic will not be captured. This impedes our understanding of the true spectrum of disease severity for neurological disorders and the identification of disease phenotypes that can help guide management and predict outcomes.

There are three main reasons why data may not be currently available in our OMOP CDM database:

1. Some data are available within the EHR or other hospital databases, but have not yet been transformed into our OMOP CDM, for example, information regarding whether an OP appointment is new or follow-up. Data like this will be targeted in future updates to the database.
2. Other data are available in hospital databases but are not yet supported by the OMOP CDM without using extensions to the current vocabulary, for example, imaging data, although this should change with the addition of extensions to the OMOP CDM.^18^
3. Other data are simply unavailable at the hospital, for example, community prescribing data, although it is hoped that these data will be incorporated into the SDE in time.

Diagnosis data for OPs are absent both from the EHR and the OMOP CDM and this presents a fundamental problem with respect to cohort definition. Much medical research relies on being able to identify patients by their diagnosis or condition and whether they have been prescribed a particular medication. The current lack of information in both of these categories for OPs presents a barrier to conducting research on this population. This was a constraint in the present study as we were unable to stratify the subcohorts by condition, and therefore the four subcohorts were very heterogeneous. The availability of OP diagnosis data would facilitate future research and enable much more detailed analyses than we have been able to perform here.

During the analysis, we found preliminary indications that IP length of stay affects the number of records per day. We noted a difference between the median number of records in subcohorts 3 and 4, both of which include IP stays. A possible explanation for this is that subcohort 3 includes more clinically stable elective patients, admitted directly onto an IP ward, whereas subcohort 4 includes patients admitted acutely unwell through the ED. Acutely unwell patients require more intensive monitoring by comparison with clinically stable patients, which may explain the higher median number of drugs, observations and measurements for this subcohort. Subcohort 3 also had substantially more IP stays of only 1 day compared with subcohort 4; again, this may be because subcohort 3 includes patients admitted electively for day case procedures, while subcohort 4 includes acutely unwell patients admitted through ED who are likely to need a longer stay. Future research should investigate this disparity further to determine whether admission acuity and/or the length of stay are driving this difference.

During the analysis, we found preliminary indications that IP length of stay affects the number of records per day. We noted a difference between the median number of records in sub-cohorts 3 and 4, both of which include IP stays. A possible explanation for this is that sub-cohort 3 includes more clinically stable elective patients, admitted directly onto an IP ward, whereas sub-cohort 4 includes patients admitted acutely unwell through the ED. Acutely unwell patients require more intensive monitoring by comparison with clinically stable patients, which may explain the higher median number of drugs, observations and measurements for this sub-cohort. Sub-cohort 3 also had substantially more IP stays of only 1 day compared with sub-cohort 4; again, this may be because sub-cohort 3 includes patients admitted electively for day case procedures, while sub-cohort 4 includes acutely unwell patients admitted through ED who are likely to need a longer stay. Future research should investigate this disparity further to determine whether admission acuity and/or the length of stay are driving this difference.

This study was limited by the absence of OP diagnostic coding which led to heterogenous subcategories, as mentioned above. This lack of coding also led to the necessity of identifying the study population by the clinician recorded as conducting the initial OP appointment. This method of identifying neurology OPs relies on the clinician being accurately recorded and of course will include patients for whom a non-neurological diagnosis was made at the appointment. If diagnoses from neurology OP appointments were to be coded, this would allow for a much more accurate identification of a study cohort. This study is also limited by the fact that this is a single centre study; however, as many practices are the same across the NHS, the results are unlikely to differ. Future research could be conducted once the OMOP CDM has been introduced to other regions to determine if the patterns we have observed here are replicated in other regions where mandated coding has not been introduced.

We have highlighted the large difference in the number of records created between different types of hospital visit and also provided opportunity to reflect on the necessity of the amount and frequency of data capture in some hospital settings. Previous research has brought into question the efficacy of capturing large amounts of routine data^19 20^ and questioned the necessity of ordering repeat tests.^21 22^ These previous studies highlighted the need to consider carefully the way in which records are made, the underlying activity that they represent and the potential to save resources and improve patient care by eliminating unnecessary testing and data capture. Our findings highlight the difference in volumes of data recorded in OP versus IP or ED settings, but further research is needed to confirm how much of this data capture is necessary and whether systems of data capture could be made more efficient.

## Conclusion

Through this study, we have shown that the OMOP CDM can be used to analyse the number and type of records captured for patients with different visit types. We found that OP visits have far less data recorded than IP stays or ED attendances. This highlights the need to improve data capture for OPs, especially with regards to conditions and drugs, as this is vital information both for the clinician when seeing a patient and also for research. The lack of data available on OPs risks limiting the opportunities for using data both to inform clinical services and research related to neurological conditions in an OP setting.

## Data Availability

No data are available.
